# Transcranial near-infrared laser improves postoperative neurocognitive disorder in aged mice *via* SIRT3/AMPK/Nrf2 pathway

**DOI:** 10.3389/fnins.2022.1100915

**Published:** 2023-01-24

**Authors:** Junying Zhong, Le Zhao, Wensi Wu, Jiawei Chen, Shangyan Yuan, Xiaojun Zhang, Zhi Wang

**Affiliations:** ^1^Department of Anesthesiology, Sun Yat-sen Memorial Hospital, Sun Yat-sen University, Guangzhou, Guangdong, China; ^2^Department of Thoracic Surgery, Qilu Hospital of Shandong University, Jinan, Shandong, China

**Keywords:** postoperative neurocognitive disorder, neuroinflammation, oxidative stress, synaptic dysfunction, SIRT3/AMPK/Nrf2 pathway

## Abstract

**Background:**

Postoperative neurocognitive disorder (PND) is a common central nervous system (CNS) complication that might increase the morbidity and mortality of elderly patients after anesthesia/surgery. Neuroinflammation, oxidative stress, and synaptic dysfunction are closely related to cognitive dysfunction, an important clinical feature of PND. Transcranial near-infrared laser (TNIL) is regarded as an effective treatment for cognitive-related diseases by improving mitochondrial function and alleviating neuroinflammation and oxidative stress damage.

**Materials and methods:**

Aged male C57BL/6 mice underwent a carotid artery exposure procedure under isoflurane anesthesia. We treated PND-aged mice for three consecutive days (4 h post-operation, 1-laser) with 810 nm continuous wave (CW) laser 18 J/cm^2^ at 120 mW/cm^2^. The post-treatment evaluation included behavioral tests, RTq-PCR, immunofluorescence, and Western blot.

**Results:**

The results demonstrated that TNIL improved PND and the levels of synaptic function-associated proteins such as post-synaptic density protein 95 (PSD95), synaptophysin (SYP), and brain-derived neurotrophic factor (BDNF). Besides, neuroinflammatory cytokine levels of tumor necrosis factor (TNF)-α and interleukin (IL)-1β as well as microglia activation and oxidative stress damage were attenuated after TNIL treatment in aged mice with PND. Further investigation suggested that TNIL relieved oxidative stress response by activating the SIRT3/AMPK/Nrf2 pathway.

**Conclusion:**

Transcranial near-infrared laser improved cognitive impairment in aged mice with PND, which may be a promising therapeutic for PND.

## 1. Introduction

Postoperative neurocognitive disorder (PND) is the functional impairment of the nervous system activities, such as memory, executive function, and language impairment in anesthesia/surgery populations (Liu J. et al., 2021). The incidence of PND is about 8.9–46.1%, which is higher in patients over the age of 65 years (Kapila et al., 2014). PND seriously affects a patient’s life quality (Gao et al., 2005). At present, the exact pathogenesis of PND remains unclear, and there is a lack of effective treatments.

Neuroinflammation, oxidative stress, and synaptic dysfunction have been testified to be involved in the pathological process of PND (Netto et al., 2018; Wang et al., 2018; Xiao et al., 2018). Studies showed that inflammatory cytokine levels of tumor necrosis factor (TNF)-α and interleukin (IL)-1β were notably increased in the hippocampus of aged mice with PND, which indicated that neuroinflammation is an important pathogenesis of PND (Subramaniyan and Terrando, 2019). Microglia, as the main source of inflammatory factor release, closely contribute to neuroinflammation (Kim and de Vellis, 2005). Anesthesia and surgical stimulation can damage the neuronal mitochondrial function and cause an imbalance of reactive oxygen species (ROS). Furthermore, it has been reported that mitochondrial oxidative stress can result in the injury of neurons that emits excessive inflammatory cytokines (Leyane et al., 2022). The continuous release of pro-inflammatory cytokines causes a concatenation of events involving oxidative stress and cognitive impairment activation (Zhang et al., 2021).

SIRT3 is an NAD^+^-dependent deacetylation enzyme that affects mitochondria energy metabolism and oxidative stress (Zhang et al., 2020). SIRT3 not only directly deacetylates SOD2 but also increases the activity of the antioxidant enzyme Nrf2 by activating AMPK to improve the antioxidant capacity (Tao et al., 2010; Park et al., 2020). Importantly, nuclear factor-erythroid 2-related factor 2 (Nrf2) is an important transcription factor regulating oxidative stress response (Staurengo-Ferrari et al., 2018). Studies found that neurological damage and cognitive impairment were accompanied by the downregulated AMPK/Nrf2 pathway (Cao et al., 2020), suggesting that the SIRT3/AMPK/Nrf2 pathway may be a target for the treatment of PND.

Transcranial near-infrared laser (TNIL; λ = 600—1,070 nm) therapy is emerging as an effective neuroprotective therapy, regardless of acute brain damage or neurodegenerative disease (Naeser et al., 2014). For example, in the traumatic brain injury (TBI) model, TNIL increases brain-derived neurotrophic factor (BDNF) and synaptogenesis (Xuan et al., 2015). In Alzheimer’s disease (AD) model, TNIL attenuates Aβ burden and cognitive impairment (Tao et al., 2021). TNIL protected mitochondrial function by enhancing the activity of cytochrome C oxidase (CCO) and increasing adenosine triphosphate (ATP) synthesis (Foo et al., 2020). TNIL can efficiently and non-invasively penetrate into the central nervous system (CNS) and provide excellent neuroprotection, such as decreasing neuronal cell apoptosis, ameliorating dendrite atrophy, and promoting nerve regeneration (Liang et al., 2012; Guo et al., 2021). Due to the positive effect of TNIL use on the brain, we established a PND model and investigated the roles and mechanisms of TNIL, to uncover a potential treatment for PND.

## 2. Materials and methods

### 2.1. Animals

**FIGURE 1:**
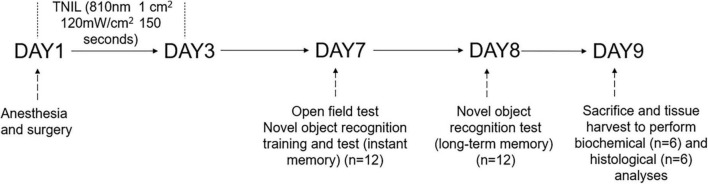
Diagram of the timeline of experimental procedures.

For this study, a total of 48 aged (18 months old, weighing 45–50 g) male C57BL/6 mice were ordered from Sun Yat-sen University (Guangzhou, China). Five mice per cage were group-housed (at 22–25^°^C) with food and water available *ad libitum* under a 12-h light/12-h dark cycle for 2 weeks to adapt to the environment. The animals were randomly assigned to four groups, namely, the CON group (no intervention), the CON + TNIL group (light therapy), the PND group (anesthesia, surgery, and sham light therapy), and the PND + TNIL group (anesthesia, surgery, and light therapy) (*n* = 12). Mice in the PND group were treated for sham light therapy with the device turned off; mice in the CON + TNIL and PND + TNIL groups received light therapy (18 J/cm^2^, 3 days). All mice heads were shaved to eliminate hair interference. In the experiment, mice received isoflurane anesthesia and surgery on day 1; TNIL treatment was performed during days 1–3; behavioral testing was performed during days 7–8 (*n* = 12); the brain was harvested to perform biochemical (*n* = 6) and histological (*n* = 6) analyses on day 9. The schematic timeline of the experimental process is shown in Figure 1.

### 2.2. PND mouse model

The right carotid artery exposure procedure was performed under isoflurane anesthesia to establish the PND model, as described in other studies (Min et al., 2022). Mice were anesthetized by 2% isoflurane and kept on spontaneous respiration during the procedure. Rectal temperature was monitored and maintained at 37^°^C with the aid of a heating blanket (69020, RWD, China). After the mouse was anesthetized by isoflurane for at least 20 min, a 1.5-cm midline neck incision and soft tissue dissection with 1-cm long right common carotid artery exposure were carefully performed without any damage to the vagus nerve. Subsequently, the wound was irrigated and closed by using a 4–0 surgical suture. The surgical procedure was performed under sterile conditions and lasted around 12 min. The total duration of general anesthesia was 2 h, and an anesthesia monitor (B450, GE, USA) was used to dynamically monitor the depth of anesthesia and maintain the anesthesia level. No response to toe pinching was observed during the whole process. After the surgery, the incision was supplied with 2.5% lidocaine cream to alleviate the postoperative pain.

### 2.3. Transcranial near-infrared laser treatment

Aged mice in the CON + TNIL and PND + TNIL groups received laser treatment 4 h after surgery for 3 consecutive days. We used an 810 nm wavelength and 1 W maximum power output diode laser (HW810AD2000-34F, Shenzhen Infrared Laser Technology Co., Ltd., China), which emitted 810 nm CW wavelength near-infrared radiation. Mice were manually restrained by holding the body and shaved head, and a laser was fixed above the midline on the back of the head in the area between the eyes and ears. The spot size was 1 cm^2^ and the distal tip of the fiber optic provided a power density of 120 mW/cm^2^. The laser irradiation was continued for 150 s, and the total energy fluence was 18 J/cm^2^. We also made a measurement of TNIL penetration across the skull of the mice, where the skull was excised from the head of the mice and placed on a foil-coated vessel with a calibrated light sensor at the bottom. Then, TNIL was irradiated to the skull, and the penetration power was recorded by the sensor (the laser was about 4 cm away from the skull, and the skull was about 3 cm away from the sensor), and the measured transmittance was about 8%. All complete parameters including device information, irradiation parameters, and treatment parameters used in TNIL treatment are reported in Table 1.

**TABLE 1:** Transcranial near-infrared laser parameters.

Device information	
Manufacturer	Shenzhen Infrared Laser Technology Co., Ltd., CHN
Model identifier	HW810AD2000-34F
Emitter type	Laser
**Irradiation parameters**	
Center wavelength	810 nm
Operating mode	Continuous wave
**Treatment parameters**	
Beam spot size	1 cm^2^
Irradiance	120 mW/cm^2^
Exposure duration	150 s
Radiant exposure	18 J/cm^2^
Number and frequency of treatment sessions	Once daily, 3 consecutive days

### 2.4. Behavioral tests

All behavioral experiments were conducted during the light phase between 8 a.m. and 6 p.m. in a sound-isolated room and an unbiased double-blind manner.

#### 2.4.1. Open field test (OFT)

After 7 days of anesthesia/surgery, each mouse was placed in a black opaque plastic chamber (60 × 60 × 50 cm, ZH-ZFT, Anhui Zhenghua Biological Instrument Equipment Co., Ltd., Anhui, China) and freely explored for 5 min. A video camera was used to automatically track and record the movement traces of the mice with the video tracking system (Smart version 3.0.06; Panlab Harvard Apparatus, Barcelona, Spain) and then analyze the total distance in the whole area and the time spent in the center area. The field was wiped with 75% ethanol after each test to avoid olfactory cues.

#### 2.4.2. Novel object recognition (NOR) test

To evaluate the mice’s momentary and long-term memory as described by Zhang et al. (2022), we conducted the novel object recognition (NOR) in an open field (60 × 60 × 50 cm) 2 h after the open field test (OFT). In the training period, two identical objects were placed at adjacent angles in the field. The mice were placed in the experimental plat with their backs turned towards the objects and allowed to explore freely for 5 min. If the total exploration time on two objects was less than 5 s, the mouse was eliminated. One of the objects was replaced by a new object 30 s and 24 h later. The mice were placed in the room with their backs turned towards the object and allowed to explore for 5 min. Animal behavior was recorded by a video tracking system (Smart version 3.0.06, Panlab Harvard Apparatus, Barcelona, Spain). The exploration time of new (T2) and old (T1) objects within 5 min was recorded, and the memory ability of the mice was quantified by the discriminant index (DI) = T2/(T1 + T2). As others described earlier (Lai et al., 2021), the DIs at 30 s and 24 h after training reflected momentary and long-term memory, respectively. During the test interval, the field was cleaned with 75% ethanol to eliminate feces and odors.

### 2.5. Brain tissue harvesting

After behavioral tests, mice were deeply anesthetized by isoflurane and transcardially perfused with normal saline, after which the brain tissue was removed (*n* = 6). The hippocampus was isolated for subsequent experiments on genes and protein levels. All dissection procedures were performed on ice and stored at −80^°^C before use. As for others, the transcardial perfusion was given with normal saline and 4% paraformaldehyde (*n* = 6). Brains were fixed in 4% paraformaldehyde solution for 24 h and then transferred to 10, 20, and 30% sucrose solution for 1 day each to dehydration. The brain tissue was embedded with optimum cutting temperature (OCT) (4583, SAKURA, JP) and then stored for subsequent immunofluorescent staining.

### 2.6. Immunofluorescent staining

The immunofluorescent labeling and quantification of the staining were similar to previous studies (Zheng et al., 2017; Lai et al., 2021). Notably, 20-μm thick sections of the coronal brain were sequentially cut from bregma -2 to -4 mm in the hippocampus of mice. The sections were washed three times with phosphate-buffered solution (PBS) to remove OCT from the surface. Subsequently, permeable liquid (PBS plus 0.3% TritonX-100) was added and the sections were allowed to stand at room temperature (RT) for 10 min. Then, the sections were washed three times with PBS. After blocking with 5% goat serum (16210072, Gibco, USA) for 30 min at RT, the sections were incubated with the primary Iba-1 antibody (1:100, 10904-1-AP, Proteintech, China) at 4^°^C overnight. Tissue sections were washed three times with PBS and then incubated with Cy3-labeled goat anti-rabbit IgG (H + L) (1:200, A0516, Beyotime, China) at RT for 2 h and then incubated with DAPI (G1012, Servicebio, China) at RT for 10 h. The tissue sections were sealed with an anti-fluorescence quenching reagent (P0128M, Beyotime, China). The images of each section were acquired by an inverted fluorescence microscope (Olympus IX73, JP). Image J (National Institutes of Health, Bethesda, MD, USA) was used to quantify the mean value of the immunofluorescence and numbers of Iba1^+^ cells in each section. For each mouse brain, six consecutive slices of the hippocampus were used for positive staining and cell counting and averaged to reflect the level of fluorescence intensity and Iba1^+^ cells. The positively stained area for the Iba1 marker was presented as a ratio of average fluorescence intensity. The numbers of Iba1- and DAPI-co-staining positive cells in the CA1 region were counted. The quantitative analyses were performed by blind method.

### 2.7. Western blot

Hippocampus was dissolved in RIPA lysis buffer (P0013B, Beyotime, China) and protein concentration was measured by a BCA protein quantification kit (P0010, Beyotime, China). The sample was electrophoresed on SDS-PAGE gels to separate protein and transferred to PVDF membranes. The membranes were blocked with 5% skim milk (A600669, Sangon Biotech, China) in Tris-buffered saline with Tween (TBST) for 2 h at RT and then incubated with primary antibodies overnight at 4^°^C. After the membranes were washed three times in TBST, they were incubated with secondary antibodies for 2 h at RT. The protein bands were detected by ECL reagent (WBKLS0100, Merck Millipore, USA), exposed to a chemiluminescence imager (SmartChemi™ 910, China), and quantitated with Image J. Each sample was subjected to Western blotting analysis using the following primary antibodies, as reported in Table 2. Horseradish peroxidase (HRP)-conjugated goat anti-rabbit IgG (1:1,000, A0208, Beyotime, China) was used as the secondary antibody.

**TABLE 2:** Primary antibodies used in the present study.

Antigen	Host	Manufacturer (catalog number)	Dilution used
TNF-α	Rabbit	AF8208, Beyotime, CHN	1:1,000
IL-1β	Rabbit	AF7209, Beyotime, CHN	1:1,000
SYP	Rabbit	AF8091, Beyotime, CHN	1:1,000
PSD95	Rabbit	AF1096, Beyotime, CHN	1:1,000
BDNF	Rabbit	AF1423, Beyotime, CHN	1:1,000
SIRT3	Rabbit	AF5303, Beyotime, CHN	1:1,000
phospho-AMPKα (Thr172) (40H9)	Rabbit	2535, Cell Signaling Technology, USA	1:1,000
AMPKα	Rabbit	2532, Cell Signaling Technology, USA	1:1,000
Nrf2	Rabbit	T55136, Abmart, CHN	1:1,000
GADPH	Mouse	T0004, Affinity, USA	1:10,000
β-tubulin	Rabbit	AF1216, Beyotime, CHN	1:1,000

### 2.8. Reverse transcription-quantitative polymerase chain reaction

Total RNA was extracted by an RNA Quick Purification kit (RN001, ES Science, China). The concentrations of the RNA samples were determined by NanoDrop ND-2000 (Thermo, USA) instrument. Reverse transcription was finished by using All-in-One First-Strand Synthesis MasterMix (with dsDNase) (F0202, LABLEAD, China). Taq SYBR^®^ Green qPCR Premix (R0202, LABLEAD, China) and Roche Light Cycler 480 II Real-Time PCR System (Roche, USA) were used for qPCR quantification. GAPDH was used as the internal reference for normalizing target gene expression. Data were obtained using the 2^–ΔΔCt^ method. The sequences of primers are presented in Table 3.

**TABLE 3:** Primer sequences for RT-PCR (mouse).

Primers for RT-PCR (5′–3′)		Sequence
Glutathione peroxidase-1 (GPX1)	Forward	CCACCGTGTATGCCTTCTCC
	Reverse	AGAGAGACGCGACATTCTCAAT
Thioredoxin-2 (TXN2)	Forward	TGGGCTTCCCTCACCTCTAAG
	Reverse	CCTGGACGTTAAAGGTCGTCA
Peroxiredoxin 3 (PRDX3)	Forward	GGTTGCTCGTCATGCAAGTG
	Reverse	CCACAGTATGTCTGTCAAACAGG
Forkhead box protein O1 (FOXO1)	Forward	CCCAGGCCGGAGTTTAACC
	Reverse	GTTGCTCATAAAGTCGGTGCT
Heme oxygenase 1 (HO-1)	Forward	AGGTACACATCCAAGCCGAGA
	Reverse	AGGTACACATCCAAGCCGAGA
NADPH oxidase 2 (NOX2)	Forward	TGTGGTTGGGGCTGAATGTC
	Reverse	CTGAGAAAGGAGAGCAGATTTCG
BTB and CNC homology 1 (BACH1)	Forward	TGAGTGAGAGTGCGGTATTTGC
	Reverse	GTCAGTCTGGCCTACGATTCT
Acyl-CoA synthetase long-chain family member 4 (ACSL4)	Forward	CTCACCATTATATTGCTGCCTGT
	Reverse	TCTCTTTGCCATAGCGTTTTTCT
Myeloperoxidase (MPO)	Forward	AGTTGTGCTGAGCTGTATGGA
	Reverse	CGGCTGCTTGAAGTAAAACAGG

### 2.9. MDA evaluation

Malondialdehyde (MDA) was measured by a Lipid Peroxidation MDA Assay Kit (S0131S, Beyotime, China) according to the manufacturer’s instructions. A microplate reader (TECAN Spark10M, China) was used to determine MDA activity by measuring the absorbance at 532 nm (U/mg of protein).

### 2.10. Statistical analysis

All data were presented as the mean ± SEM. Statistical analyses were performed using GraphPad Prism version 7.0 (GraphPad Software, Inc.). The inter-group comparisons were analyzed by one-way ANOVA with a Tukey *post hoc* test for multiple comparisons. The data of NOR were analyzed by the Kruskal–Wallis test and *post hoc* comparisons were conducted by Dunn’s test. A statistically significant difference was defined as *p* < 0.05.

## 3. Results

### 3.1. TNIL improved cognitive function in aged PND mice

**FIGURE 2:**
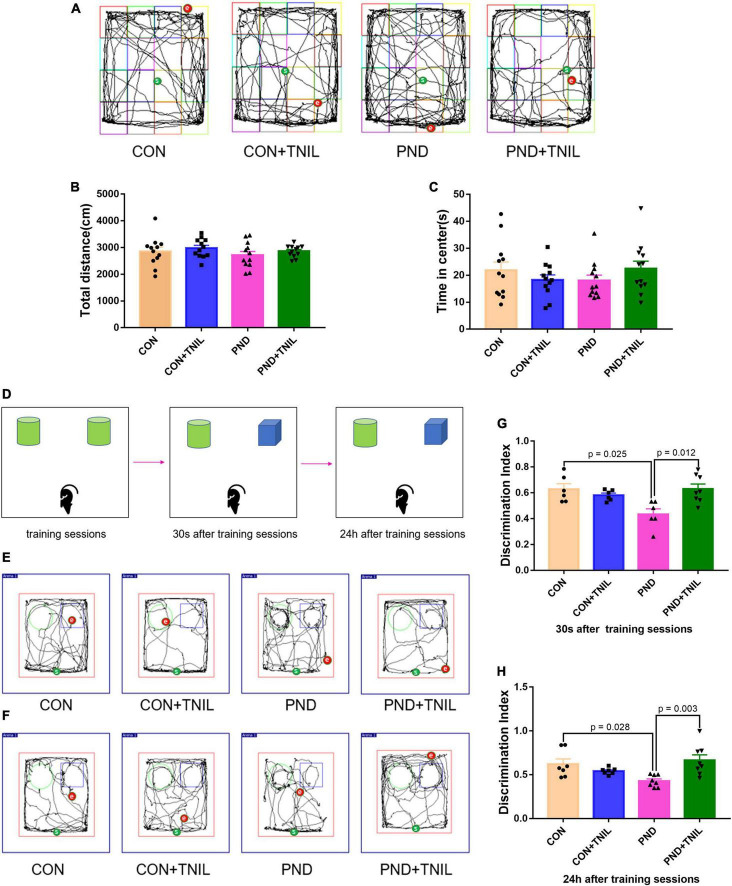
Transcranial near-infrared laser alleviated anesthesia-/surgery-induced cognitive dysfunction in aged mice. **(A)** Representative movement traces of OFT. **(B)** The total distance among four groups in OFT. **(C)** Time spent in the center among four groups in OFT. **(D)** The schematic diagram of NOR. **(E,F)** The representative movement traces at 30 s and 24 h after training sessions of NOR. **(G,H)** Performance in NOR. Data are shown as mean ± SEM (*n* = 12).

At 7 days after surgery, we assessed the locomotor activity and exploratory behavior among four groups by OFT. Mice in all groups had no significant difference in total distance and time spent in the center of OFT (Figures 2A–C). The results suggested that locomotor activity and postoperative anxiety behavior were not affected. Based on the nature of exploring new things in rodents, the spatial memory ability of mice was tested by the NOR experiment. Mice were tested for instant and long-term memory, respectively, 30 s and 24 h after the training sessions. The CON group was not significantly different from the CON + TNIL group in DI either 30 s or 24 h after training sessions. Then, we found that the DIs were decreased in the PND group compared with the CON group, and TNIL improved the DIs at 30 s and 24 h (Figures 2D–H). In other words, these results indicated that anesthesia/surgery induced instant and long-term memory dysfunctions that were attenuated by TNIL in aged mice. In addition, these data showed that TNIL had no significant effect on the behavior of mice in the CON + TNIL group. Therefore, we conducted subsequent experiments in the CON, PND, and PND + TNIL groups to investigate the possible mechanism and signaling pathway.

### 3.2. TNIL attenuated neuroinflammation in aged PND mice

**FIGURE 3:**
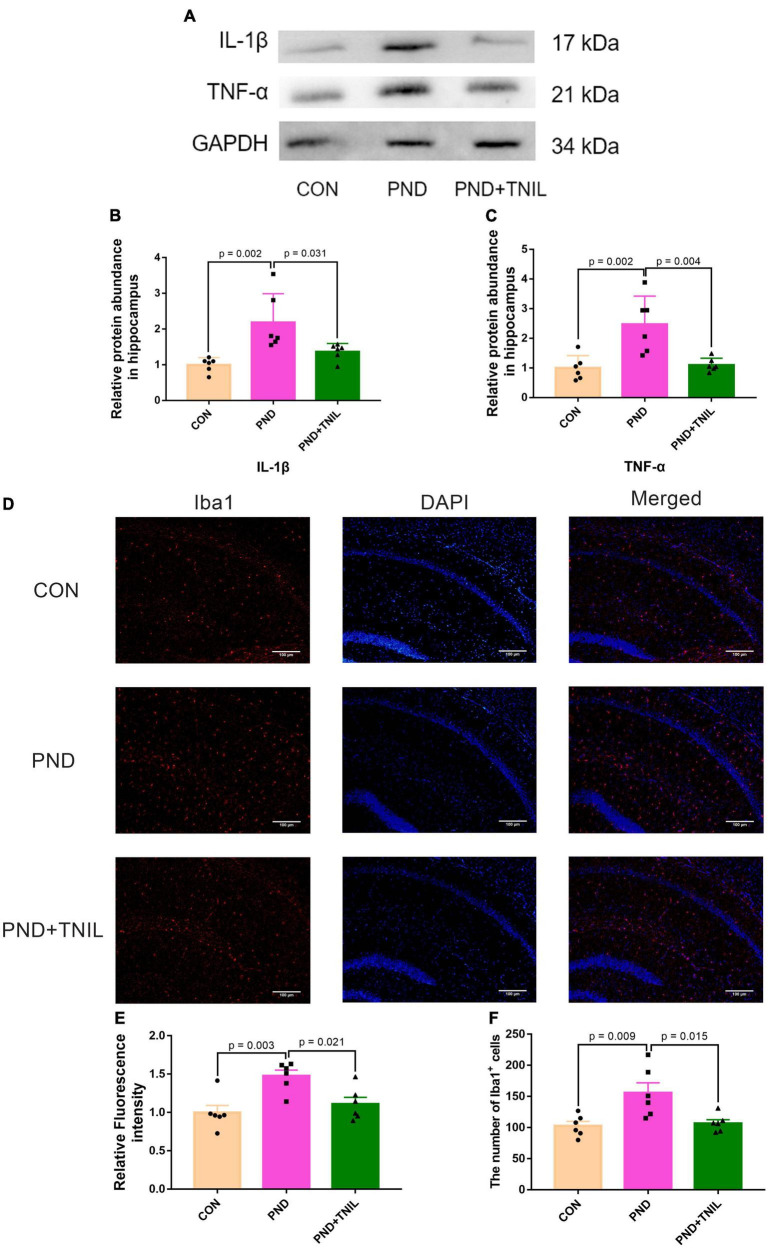
Transcranial near-infrared laser alleviated hippocampus neuroinflammation of PND mice. **(A)** Representative Western blots of IL-1β and TNF-α. **(B,C)** Relative protein expressions of IL-1β and TNF-α, normalized to that of the GADPH internal control. **(D)** Representative images of Iba1 in the hippocampal CA1 region. **(E)** Quantification of Iba1 relative fluorescence intensity. **(F)** Quantitative analysis of Iba1^+^ cells. Scale bar = 100 μm. Data are shown as mean ± SEM (*n* = 6).

The IL-1β and TNF-α were employed to assess the levels of neuroinflammation. Compared with the CON group, the expressions of IL-1β and TNF-α were significantly upregulated in the PND group. Furthermore, the expressions were downregulated in the PND + TNIL group compared with the PND group (Figures 3A–C). Given the proinflammatory factors are mainly produced by activated microglia, we studied the activated microglial marker Iba1 by immunofluorescence staining. The results showed the Iba1 expression and the number of Iba1^+^ cell increased in the PND group compared with the CON group, and TNIL attenuated the increase (Figures 3D–F). These results suggested that the neuroinflammation and microglia activation of the hippocampus of aged mice with PND were attenuated by TNIL.

### 3.3. TNIL alleviated oxidative stress imbalance in aged PND mice

**FIGURE 4:**
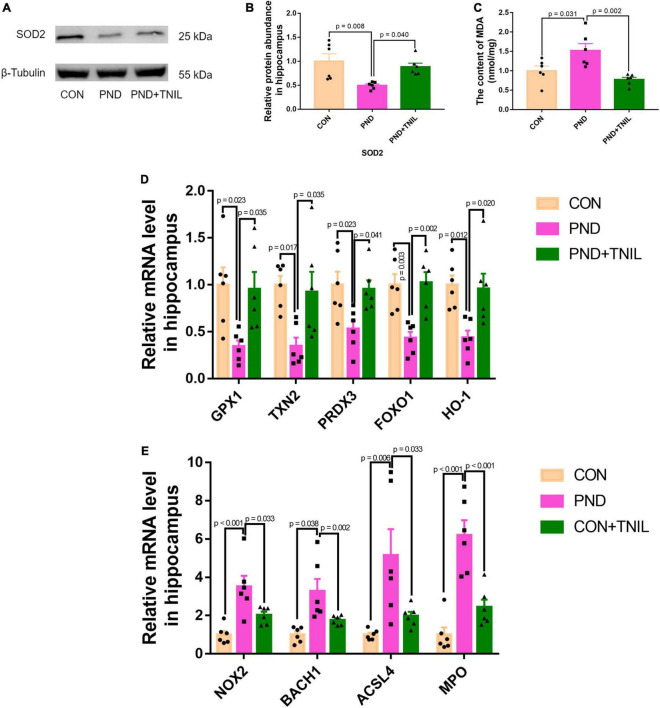
Transcranial near-infrared laser alleviated hippocampal oxidative stress of PND mice. **(A)** Representative Western blot images of SOD2. **(B)** Relative protein expression of SOD2. **(C)** The content of MDA in the hippocampus of mice. **(D,E)** Antioxidant genes and oxidant genes mRNAs expression measured by RT-qPCR. The abundance of each mRNA of interest was expressed relative to the abundance of GAPDH-mRNA, as an internal control. Data are shown as mean ± SEM (*n* = 6).

To research the oxidative stress response of aged mice with PND, we first detected the levels of antioxidant enzyme SOD2 and lipid peroxidation product MDA. The results showed a decreased protein expression of SOD2 but an increased content of MDA existed in the PND group, which was relieved in the PND + TNIL group (Figures 4A–C). Then, we tested the expression of antioxidant- and oxidant-related genes in the hippocampus among the three groups. It was found that the mRNA levels of these antioxidant genes (GPX1, TXN2, PRDX3, FOXO1, and HO-1) were decreased in the PND group, and TNIL reversed these changes (Figure 4D). Conversely, compared with the CON group, the mRNA levels of oxidant genes (NOX2, BACH1, ACSL4, and MPO) were significantly increased in the PND group, while TNIL significantly attenuated these upregulated genes (Figure 4E). These results indicated that oxidative stress response may be closely involved in the pathogenesis of PND, and TNIL can significantly reduce oxidative stress imbalance.

### 3.4. TNIL improved hippocampal synaptic dysfunction of aged PND mice

**FIGURE 5:**
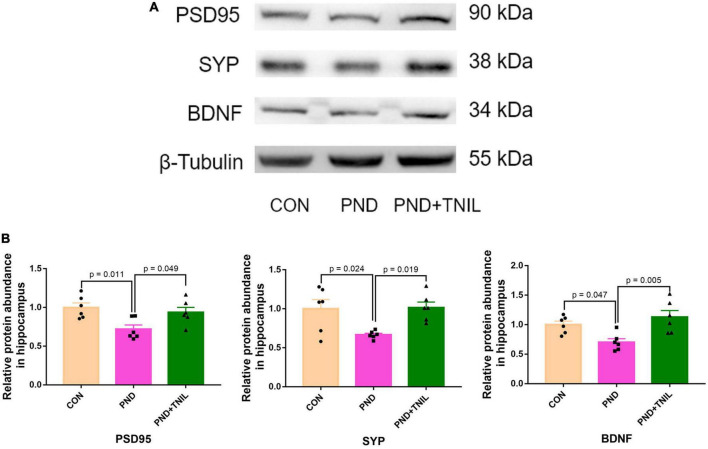
Transcranial near-infrared laser improved hippocampal synaptic dysfunction of aged mice with PND. **(A)** Representative Western blots of PSD95, SYP, and BDNF in the hippocampus. **(B)** Relative protein expression of PSD95, SYP, and BDNF in the hippocampus. Data are shown as mean ± SEM.

The normal synaptic function is considered to be the biological basis of learning and memory (Shivarama Shetty and Sajikumar, 2017). BDNF is the crucial regulator of synaptic function, which promotes synaptogenesis and synaptic transmission (Waterhouse and Xu, 2009). Western blot results showed that the expressions of PSD95, SYP, and BDNF were reduced in the PND group compared with the CON group while increased in the PND + TNIL group (Figures 5A, B). These data indicated that TNIL may have a protective effect against hippocampal synapse impairment in aged mice with PND.

### 3.5. TNIL improved PND *via* adjusting SIRT3/AMPK/Nrf2 pathway

**FIGURE 6:**
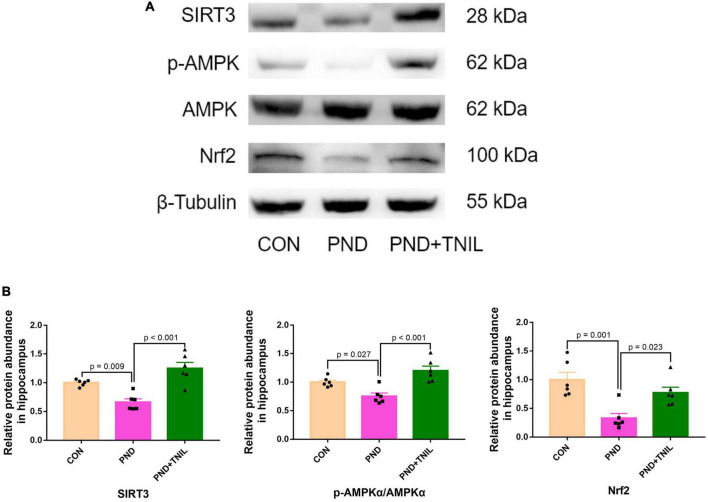
Transcranial near-infrared laser activated SIRT3/AMPK/Nrf2 pathway. **(A)** Representative Western blots of SIRT3, p-AMPK, AMPK, and Nrf2. **(B)** Relative protein expression of SIRT3, p-AMPK, AMPK, and Nrf2. Data are shown as mean ± SEM (*n* = 6).

To reveal whether the balance of SIRT3/AMPK/Nrf2 was affected by TNIL, we carried out Western blotting to measure the expression levels of SIRT3, p-AMPK, AMPK, and Nrf2 proteins. The results showed that the expression of SIRT3, p-AMPK/AMPK, and Nrf2 was significantly decreased in the PND group compared with the CON group while increased in the PND + TNIL group (Figures 6A, B). These results suggested that the SIRT3/AMPK/Nrf2 pathway might participate in TNIL treatment of PND-aged mice.

## 4. Discussion

The postoperative neurocognitive disorder is a common CNS complication in patients after anesthesia/surgery, especially in aged people, which causes a negative effect on personality, social ability, and cognitive function (Evered et al., 2018; Liu J. et al., 2021). Increasing evidence showed that neuroinflammation, oxidative stress damage, and synaptic dysfunction could be responsible for PND (Zhao et al., 2019; He et al., 2022). Consequently, alleviating these mechanisms may provide a potential method for the treatment of PND.

Transcranial near-infrared laser has shown positive results in the treatment of stroke, TBI, and neurodegenerative diseases (Oron et al., 2006; Xuan et al., 2015; Lu et al., 2017). TNIL at a wavelength of 810 nm can effectively and non-invasively penetrate into the skull and brain tissue (Streeter et al., 2004). It was reported that 810 nm laser improved cognitive function and increased BDNF and synaptogenesis in TBI mice. Besides, studies showed that the most effective treatment was 3 consecutive days after surgery (Xuan et al., 2014). Our study showed that TNIL improved spatial learning and memory in aged mice with PND and had no influence on the normal group. The results indicated that TNIL improved cognitive function in aged PND mice.

A main mechanism of TNIL was absorbed by CCO, further increased ATP production, inhibited neuroinflammation, and improved antioxidant activity (Lapchak and De Taboada, 2010; Lee et al., 2017). The microglia activation secreted inflammatory cytokines including IL-1β and TNF-α, causing impaired synaptic plasticity, which further induced cognitive dysfunction (Xiao et al., 2018; Liu Y. et al., 2021). TNIL decreased the levels of TNF-α and IL-1β that have been reported in TBI mice (Salehpour et al., 2019). We found that massive activation of microglia accompanied by the levels of the inflammatory mediators including TNF-α and IL-1β was significantly increased in the hippocampus of PND mice, and TNIL attenuated these changes. These results suggested that TNIL ameliorated the anesthesia-/surgery-induced microglial activation and neuroinflammation response.

Oxidative stress is defined as an imbalance between pro-oxidants and antioxidants, which is a crucial step in the mechanisms involved in PND (Patel, 2016). A previous study has reported that the oxidative damage to lipids was enhanced and antioxidant enzyme SOD2 activity was decreased in the PND model (Netto et al., 2018). In the present study, we found that anesthesia and surgery induced upregulation of MDA and downregulation of SOD2 expression in PND mice, and TNIL could reverse the above phenomenon. While the mRNA expressions of antioxidant genes were decreased, those of oxidant genes were increased in the hippocampus of aged mice with PND, which were also reversed after the treatment with TNIL. These results demonstrated the role of TNIL in the benefit of reducing oxidative stress damage.

Synapses are the key parts for functional connection and information transmission between neurons (Park and Goda, 2016). ROS is regarded as a major regulator of synaptic function and growth, and its reduction prevents the age-related decline in long-term potentiation (LTP) (Milton and Sweeney, 2012; West and Sweeney, 2012). In addition, neuroinflammation and oxidative stress influence neurotrophic factors, especially BDNF, which can increase synaptic plasticity and neurogenesis (Gardiner et al., 2009; Wang et al., 2020). Our previous studies have shown that the lower level of the synaptic proteins, i.e., PSD95, SYP, and BDNF, is closely related to cognitive impairment (Wu et al., 2021). In the present study, we found that the expression levels of SYP, PSD95, and BDNF were downregulated in the hippocampus of PND mice, and TNIL treatment ameliorated these phenomena. The result suggested that TNIL has a positive therapeutic effect on PND *via* improving synaptic function.

However, the mechanisms underlying the antioxidant effect after TNIL treatment are still unclear. TNIL corresponds to light absorption by CCO and causes more electron transfer through the mitochondrial respiratory chain, accelerating NADH transformation into NAD^+^ (Salehpour et al., 2017; Heinig et al., 2020). Previous experiments showed that increased nicotinamide adenine dinucleotide^+^ (NAD^+^)/nicotinamide adenine dinucleotide (NADH) ratio improved SIRT3 activity and thus alleviated oxidative stress (Karamanlidis et al., 2013; Mathieu et al., 2016; Ren et al., 2017). Sirtuin3 (SIRT3) is a class III histone deacetylase (HDAC) predominantly located in mitochondria and plays an important role in oxidative protection and neuroinflammation in cognitive-related diseases (Ansari et al., 2017; Jiang et al., 2017; Liu Q. et al., 2021). AMPK is a key molecule in biological energy regulation and also could reduce ROS (Hou et al., 2019; Deng et al., 2020). SIRT3 directly affects AMPK activity *via* phosphorylation (Lee et al., 2019). The antioxidant effect of the upregulation of SIRT3-dependent AMPK expression has also been reported (Xin and Lu, 2020). Moreover, AMPK could also modulate oxidative stress through the regulation of Nrf2-mediated phase II antioxidant enzymes including SOD and catalase, thus reducing the cell damage caused by ROS and electrophiles (Zimmermann et al., 2015; Lv et al., 2019). Attenuated oxidative stress *via* activating the AMPK/Nrf2 pathway has been proposed in rats after subarachnoid hemorrhage (SAH) (Huang et al., 2022). In our study, TNIL significantly increased SIRT3/AMPK/Nrf2 signaling expression in the hippocampus of PND mice and demonstrated that the SIRT3/AMPK/Nrf2 pathway may be the target of TNIL-induced antioxidant effect.

However, there are several limitations to our study. First, only 7 days of cognitive performance by NOR after anesthesia/surgery was evaluated. It is expected that the detection of the effect in the medium term (1–3 months) or longer in a future study. Second, we will intensively study the relationship between SIRT3/AMPK/Nrf2 pathway and oxidative stress in the PND model by using the SIRT3 inhibitor (3-TYP) in the follow-up studies. Third, the synaptic function is not perfect, and we will add Golgi staining and transmission electron microscopy experiment to observe the form of dendrites, dendritic spines, and synapses of neurons in the later experiment. Finally, TNIL improved PND in aged mice, but whether it is effective in other animal models or patients remains unclear, which needs to be confirmed in future studies.

## 5. Conclusion

The current study indicated that TNIL attenuated PND by improving the anesthesia- and surgery-induced neuroinflammatory, oxidative stress response, and synaptic dysfunction in the hippocampus *via* activating the SIRT3/AMPK/Nrf2 pathway. TNIL may provide a new and effective countermeasure for the treatment of PND in aged patients.

## Data availability statement

The original contributions presented in this study are included in the article/supplementary material, further inquiries can be directed to the corresponding authors.

## Ethics statement

The animal study was reviewed and approved by the animal ethics committee of Sun Yat-sen University (Approval No: SYSU-IACUC-2022-000972).

## Author contributions

ZW and XZ conceived and designed the study. JZ and LZ carried out the tests. JZ and WW performed the statistical analysis and drafted the manuscript. JC and SY supervised the project. All authors read and approved the final submission.
